# Acquired bilateral facial palsy: a systematic review on aetiologies and management

**DOI:** 10.1007/s00415-023-11897-7

**Published:** 2023-07-31

**Authors:** Giulia Molinari, Daniela Lucidi, Ignacio Javier Fernandez, Alice Barbazza, Elena Vanelli, Federico Lami, Gaia Federici, Cecilia Botti, Livio Presutti, Roberto D’Angelo, Rita Rinaldi, Matteo Alicandri-Ciufelli

**Affiliations:** 1 Department of Otolaryngology - Head and Neck Surgery, IRCCS Azienda Ospedaliero-Universitaria di Bologna, Bologna, Italy; 2 Department of Medical and Surgical Sciences (DIMEC), Alma Mater Studiorum - Università di Bologna, Bologna, Italy; 3 Department of Otolaryngology - Head and Neck Surgery, Azienda Ospedaliero-Universitaria Policlinico di Modena, University of Modena and Reggio Emilia, Modena, Italy; 4 Department of Biomedical and Neuromotor Sciences (DIBINEM), Alma Mater Studiorum - Università di Bologna, Bologna, Italy; 5 IRCCS Istituto Scienze Neurologiche di Bologna, Bologna, Italy

**Keywords:** Bilateral facial palsy, Acquired facial palsy, Facial paralysis, Facial nerve, Facial function

## Abstract

**Objective:**

To systematically review the published cases of bilateral facial palsy (BFP) to gather evidence on the clinical assessment and management of this pathology.

**Methods:**

Following PRISMA statement recommendations, 338 abstracts were screened independently by two authors. Inclusion criteria were research articles of human patients affected by BFP, either central or peripheral; English, Italian, French or Spanish language; availability of the abstract, while exclusion criteria were topics unrelated to FP, and mention of unilateral or congenital FP. Only full-text articles reporting the diagnostic work-up, the management, and the prognosis of the BFP considered for further specific data analysis.

**Results:**

A total of 143 articles were included, resulting a total of 326 patients with a mean age of 36 years. The most common type of the paralysis was peripheral (91.7%), and the autoimmune disease was the most frequent aetiology (31.3%). The mean time of onset after first symptoms was 12 days and most patients presented with a grade higher than III. Associated symptoms in idiopathic BFP were mostly non-specific. The most frequently positive laboratory exams were cerebrospinal fluid analysis, autoimmune screening and peripheral blood smear, and the most performed imaging was MRI. Most patients (74%) underwent exclusive medical treatment, while a minority were selected for a surgical or combined approach. Finally, in more than half of cases a complete bilateral recovery (60.3%) was achieved.

**Conclusions:**

BFP is a disabling condition. If a correct diagnosis is formulated, possibilities to recover are elevated and directly correlated to the administration of an adequate treatment.

## Introduction

Bilateral facial paralysis (BFP) is a rare entity, representing 0.3−2% of all facial palsies. It occurs in both paediatric and adult patients and can have a congenital or acquired cause among neurologic, idiopathic, infectious, neoplastic, traumatic, iatrogenic or metabolic disorders [1]. Simultaneous palsy is defined as the involvement of the opposite side within 30 days from the onset of the first side, whereas recurrent alternated palsy is referred to a contralateral facial palsy coming later than 30 days as the first involved hemi-face [2].

In contrast with unilateral facial palsy, in the majority of BFP cases it is possible to define the etiology and, given the severity of some diseases presenting with BFP and their potential benefit from specific treatment, it is mandatory to set up a correct differential diagnosis. BFP represents an extremely disabling condition for the patient, whose specific and timely treatment may significantly increase the chance of recovery.

Several case reports and case series of BFP have been reported in the literature, with extremely variable aetiologies. However, a comprehensive review regarding epidemiology, clinical presentation, diagnostic flowchart, treatment strategies and prognosis of this entity is lacking.

The aim of this study was to systematically review the published cases of BFP to gather evidence on the clinical assessment and management of this disabling pathology. An algorithm for the differential diagnosis and treatment of BFP is also provided to support healthcare practitioners from different fields in the management of these patients, with the aim of reducing misdiagnosis and increasing the chance of recovery through appropriate treatment.

## Methods

This systematic review was conducted following the PRISMA statement recommendations [3]. The following search string was run on PubMed, Scopus, Medscape, Ovid databases: (infectious[All Fields] OR autoimmune[All Fields] OR acquired[All Fields] OR neoplastic[All Fields] OR traumatic[All Fields] OR metabolic[All Fields]) AND (bilateral[All Fields] OR (recurrent[All Fields] AND contralateral[All Fields])) AND ("facial paralysis"[MeSH Terms] OR ("facial"[All Fields] AND "paralysis"[All Fields]) OR "facial paralysis"[All Fields] OR ("facial"[All Fields] AND "palsy"[All Fields]) OR "facial palsy"[All Fields]).

After running the above search string in January 2023 and duplications removal, the 338 titles and abstracts obtained were screened independently by two of the authors (MG, LF), who subsequently met and discussed disagreements on citation inclusion. Inclusion criteria for citations were research articles of human patients affected by BFP, either central or peripheral; English, Italian, French, or Spanish language; availability of the abstract. Exclusion criteria were topics unrelated to facial palsy, and mention of unilateral or congenital facial palsy.

Afterwards, the full-text articles identified underwent a second screening by the same two authors. Full texts were considered regardless of their study design, in order not to miss any relevant data, and were included if BFP cases were confirmed. Types of BFP included in this review were: simultaneous bilateral palsy, defined as the development of facial palsy involving the initially spared side within four weeks after onset of first-side palsy (with the paralysis not necessarily being sudden or complete on both sides), and recurrent alternating palsy, consisting in recurrence of palsy involving the opposite side (at least one episode on one side and one episode on the opposite side, in the same patient) [2, 4].

**Fig. 1:**
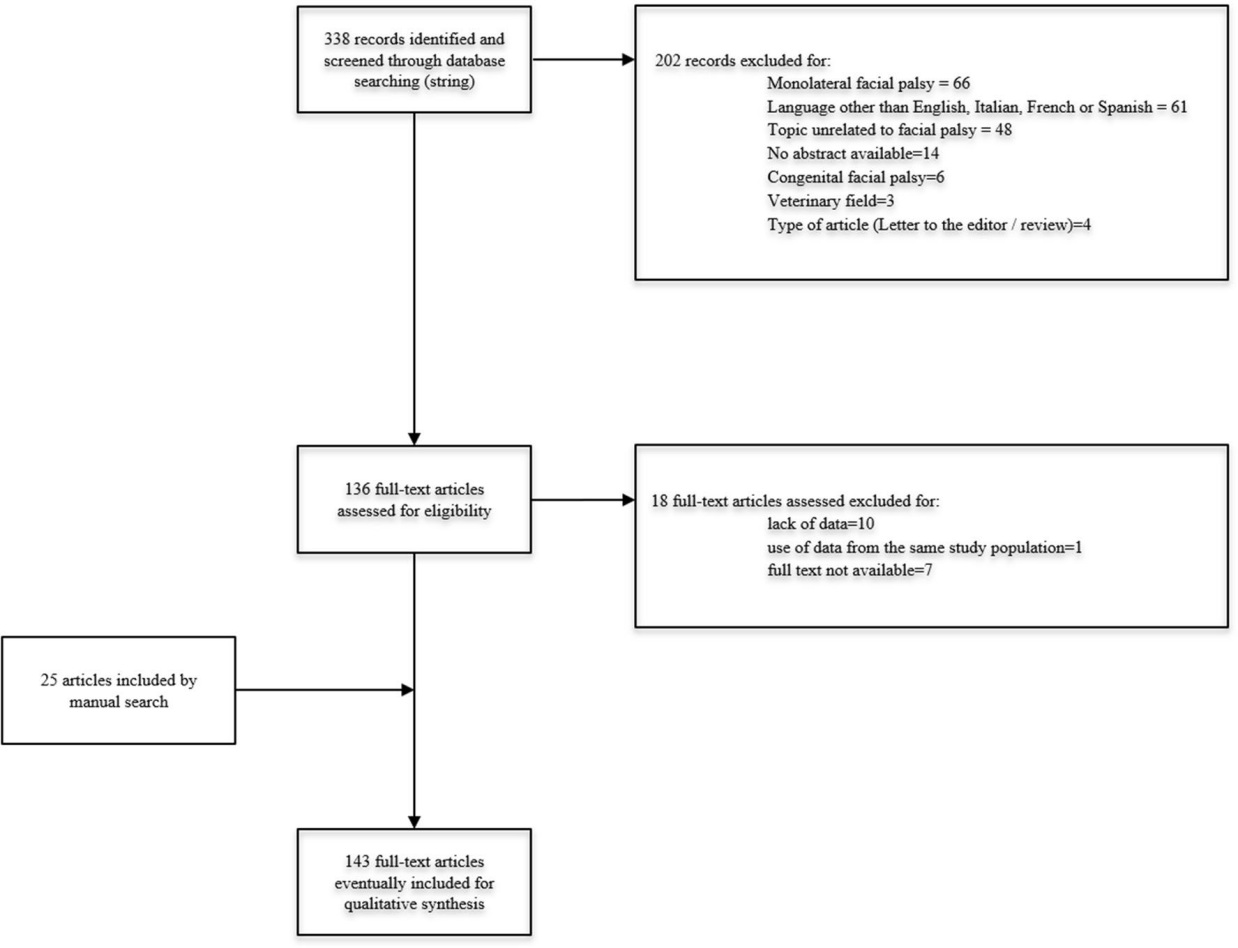
Flow chart for study selection according to PRISMA guidelines

Both treated and untreated patients were included for the data analysis. Articles reporting information on a previously published case series or whose full-text versions were not available were excluded. A further manual check of the references included in the articles was performed and the final number of articles included in the present review was defined. The flowchart of the selection process is described in Fig. 1.

The general results regarding information on patient's characteristics, clinical presentation and aetiology were extracted from the articles and recorded on an Excel database.

After the review of the included studies, only full-text articles reporting the diagnostic work-up, the management, and the prognosis (complete, partial or no recovery) of the facial paralysis for each patient were considered for further specific data analysis. Regarding the grade of facial palsy, the highest grade reached throughout the clinical course was reported as facial score at clinical presentation. There was no funding source for this study.

## Results

### General results

Running the above search string in the selected databases, 338 articles were identified. After initial check, full-text retrieval, and manual crosschecking of the references, 143 articles were eventually included for general results analysis. Articles included were published between 1977 and 2022. Most of the included articles were case reports (116) or case series (15), 10 studies were retrospective, while 2 prospective.

The total number of patients included in the qualitative synthesis was 326, with the largest study population consisting of 42 patients [5]. The mean age of affected patients was 36 (range: 7 months–93 years; standard deviation: 20). The population consisted of 146 male and 104 female patients, with a male–female ratio of 1.5:1. Gender was not reported in 76 cases.

Regarding the type of the paralysis, the majority had a peripheral FP (229 patients, 91.7%), while only 27 patients (8.3%) had a central paralysis.

The most common etiologic category was autoimmune disease (102 patients, 31.3%), followed by infectious (90 patients, 27.6%), idiopathic (51 patients, 15.6%), traumatic (28 patients, 8.6%), neoplastic (17 patients, 5.2%), iatrogenic, metabolic (3 patients each, 0.92%), and vascular disease (2 patients < 1%), as detailed in Table 1.

**Table 1:** Summary of all possible aetiologies of bilateral facial palsy

Aetiologic category	N° OF PTS	% OF PTS
**Autoimmune**	110	33.74
GBS	72	22.08
Chronic inflammatory polyneuropathy	11	3.37
Wegener's Granulomatosis	7	2.15
Miller Fisher Syndrome	7	2.15
Melkersson–Rosenthal Syndrome	4	1.23
Sjögren's syndrome	2	0.61
MPO-related vasculitis	2	0.61
Myasthenia gravis	2	0.61
Kawasaki's disease	1	0.31
Acute disseminated encephalomyelitis due to herb assumption	1	0.31
Systemic lupus erythematosus	1	0.31
**Infectious**	90	27.61
Borrelia Burgdorferi infection	43	13.19
HIV infection	11	3.37
EBV infection	6	1.84
VZV	5	1.53
Syphilis	2	0.61
Masked mastoiditis	2	0.61
Tick-borne meningitis	2	0.61
Mycobacterium leprae infection	2	0.61
Botulism	1	0.31
Cerebral toxoplasmosis in HIV + patient	1	0.31
Cryptococcal meningitis	1	0.31
Enterovirus d68 infection	1	0.31
HEV infection	1	0.31
HIV and HSV2 infection	1	0.31
HSV infection	1	0.31
Human granulocytic Ehrlichiosis	1	0.31
Japanese encephalitis	1	0.31
Leptospirosis	1	0.31
Middle ear infection by MRSA	1	0.31
Mycoplasma pneumoniae infection	1	0.31
Plasmodium malariae infection	1	0.31
Scrub Typhus infection	1	0.31
Syphilis/Tuberculoid leprosy	1	0.31
Tuberculous meningitis	1	0.31
Acute otitis media	1	0.31
**Idiopathic**	51	15.64
Bell's Palsy	50	15.34
Familial Bell's Palsy	1	0.31
**Traumatic**	28	8.59
Bilateral TB fracture	22	6.75
Head trauma	4	1.23
Bilateral condylar and/or mandibular fractures	2	0.61
**Miscellaneous**	23	7.06
Foix–Chavany–Marie syndrome	8	2.45
Neurosarcoidosis	5	1.53
Multiple cranial neuropathy	3	0.92
Brainstem encephalitis	2	0.61
Benign intracranial hypertension	1	0.31
Bulbospinal neuronopathy	1	0.31
Cogan syndrome + Borrelia burgdorferi infection	1	0.31
Osteopetrosis	1	0.31
Cholesteatoma	1	0.31
**Neoplastic**	16	4.91
Leukemia	7	2.15
Lymphoma	3	0.92
Pontine glioma	2	0.61
Epidermoid cancer	1	0.31
Ependymoma	1	0.31
Metastatic breast carcinoma to the bilateral parotid glands	1	0.31
Cancer of unknown primary	1	0.31
**Iatrogenic**	3	0.92
Transparotid approach for bilateral condylar fracture	1	0.31
Coagulation disturbances caused by antileukemic treatment	1	0.31
Acute methotrexate encephalopathy after intraventricular MTX for lymphatic leukemia	1	0.31
**Metabolic**	3	0.92
Diabetes	2	0.61
Renal osteodistrophy	1	0.31
**Vascular**	2	0.61
Pontine hemorrage	1	0.31
Basilar artery occlusion	1	0.31

*PTS* patients, *GBS* Guillain–Barrè syndrome, *MPO* myeloperoxidase, *HIV* Human immunodeficiency virus, *EBV* Epstein–Barr virus, *VZV* Varicella zoster virus, *HEV* Hepatitis E virus, *HSV* Herpes simplex virus, *MRSA* Methicillin-resistant *Staphylococcus aureus*, *TB* temporal bone, *MTX* methotrexate

As concerns the time of onset of the paralysis, in 286 patients (87.7%) the pattern was acute and simultaneous, in 31 cases (9.5%) recurrent alternating. In the remaining 9 patients (2.8%) the pattern was not reported.

### Data analysis

Considering the full-text articles selected for further specific data analysis, 122 articles and 169 cases were analysed. Among these, the mean time of onset of the paralysis after first symptoms or a trigger event was 12 days (range 0–120). In more than half of cases (105/169, 62.1%), the grade of facial palsy at onset was not reported. Considering the few studies reporting it, 52 patients (30.8%) presented with severe BFP, defined as grade higher than III according to House–Brackmann scale (H&B) on both sides, while only 3 patients (1.8%) had a mild BFP, being equal or inferior to grade III on both sides. Interestingly, 8 patients (5.1%) presented with an asymmetrical FP with a difference of more than two scores of the H&B scale.

### Associated signs and symptoms

In idiopathic BFP associated symptoms were rare, mild and non-specific, such as headache, appetite loss, vomiting, back pain, and sore throat.

On the contrary, in most of other categories (autoimmune, post-traumatic and infectious), signs and symptoms concomitant to BFP were common, often severe, and overlapping among the different types of palsy. A complete overview of associated signs and symptoms is summarized in Table 2, according to the main aetiological classifications.

**Table 2:** Associated sign and symptoms in autoimmune, infectious and traumatic forms of bilateral facial palsy (BFP)

Anatomical district	Signs and symptoms	Type of BFP		
		Autoimmune	Infectious	Trauma
General	Fatigue, drowsiness Fever and chills Myalgia, arthralgia Weight loss Back pain Nausea	X	X	
	Headache	X	X	X
	Lymphoadenopathies		X	
	Loss of consciousness			X
ENT	Agesusia-dysgeusia Hearing loss Otalgia Odynophagia	X	X	X
	Dysphagia Dysphonia Vertigo Nystagmus	X	X	
	Otorrhagia Epistaxis CSF leak TMJ typical syndrome			X
Cranial	Trigeminal neuralgia	X		
	V motor palsy			X
	Radicular pain Meningeal signs		X	
	Amnesia Diabetes insipidus Carotid-cavernous fistula			X
PNS	Motor polyneuropathy Limbs hypoesthesia-paresthesia Limbs hyporeflexia-areflexia Ataxia	X		
Ocular	Diplopia/ophtalmoplegia Xerophthalmia	X	X	X
	Blurred ision/hypovision		X	X
GI and GU	Oliguria Jaundice Epato-splenomegaly		X	
	Vomiting Diarrhea Abdominal pain Xerostomia Dark urine	X	X	
Skin	VZV skin lesion Migrant rush Maculopapular rash Petechial skin bruising		X	
CV and pulmonary	Myocarditis Pericarditis Pneumonia		X	
	Dry cough Respiratory distress	X	X	

*ENT* ear, nose, throat; *CNS* central nervous system, *CSF *cerebrospinal fluid, *TMJ* temporo-mandibular joint, *PNS* peripheral nervous system, *GI and GU* gastrointestinal and genitourinary, *VZV* varicella zoster virus, *CV* cardiovascular

### Laboratory exams and imaging

The most frequently positive laboratory exams were cerebrospinal fluid analysis (white blood cell, IgG–IgM, protein and glucose levels), autoimmune screening and peripheral blood smear.

Regarding imaging, at least one radiological investigation was performed in 126 patients (126/169 patients, 74.5%), of which 71 (56%) revealed positive findings.

The most performed imaging was brain and spine MRI (79/169 patients, 46.7%), but the most sensitive exam was temporal bone CT scan, which showed alterations in 78.3% of cases. Data are summarized in Table 3.

**Table 3:** Radiological investigations performed in bilateral facial palsy patients and relative results

Radiological investigation	N° of performed investigations	N° of investigations with positive findings (%)
Brain-spine MRI	79	40 (51%)
Head CT	52	24 (46%)
Chest X-ray	26	6 (23%)
Temporal Bone CT	23	18 (78.2%)
Other	15	9 (60%)
Skull X-ray	11	3 (27.2%)
Chest CT	11	4 (36.4%)
Abdominal CT	4	1 (25%)
Total Body CT	3	1 (33.3%)

*CT* computed tomography, *MRI* magnetic resonance imaging

Among other investigations were electrophysiological studies (electromyography, electroneuronography, Blink reflex test) in 60 patients (35.5%); audiometric tests (pure tone audiometry, auditory brainstem response, tympanometry) in 19 patients (11.2%), biopsies at different sites (according to specific clinical suspicion) in 10 patients (6%), and electroencephalography in 5 patients (3%).

**Fig. 2:**
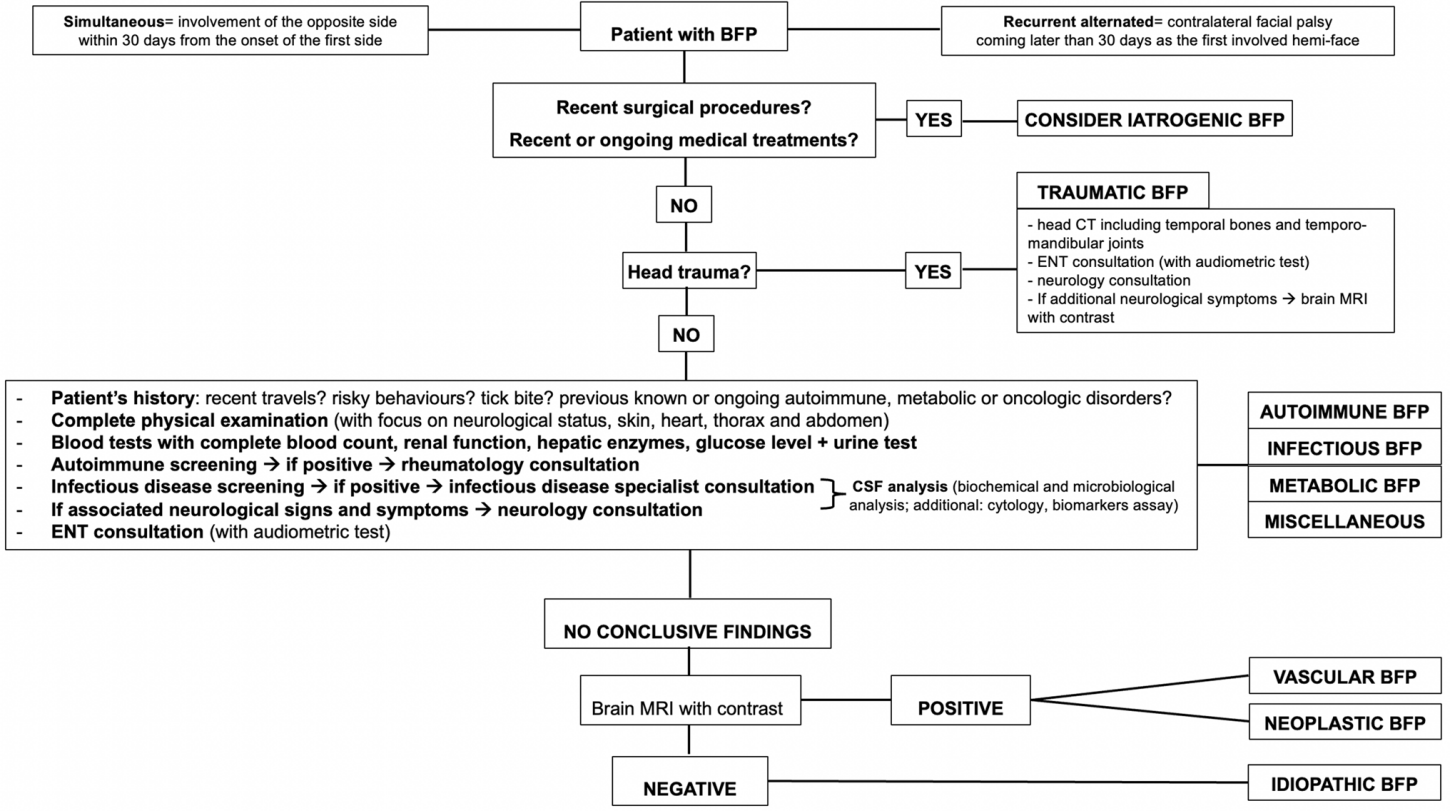
Diagnostic flow-chart for patients presenting with bilateral facial palsy (BFP). *CT* computed tomography, *ENT* ear nose thorat specialist, *CSF* cerebrospinal fluid, *MRI* magnetic resonance imaging

According to the gathered evidence regarding BFP, a flow-chart is proposed to guide physicians in diagnostic work-up of BFP (Fig. 2).

### Treatment and prognosis

Among the 169 selected patients, 125 (74%) underwent exclusive medical treatment, while a minority of patients were selected for surgical approach (3.5%) or combined medical and surgical treatment (5%). Table 4 summarizes the treatment options found in the review. About 17% of BFP cases did not receive any treatment. Only in 13 cases, rehabilitation was reported among the management options.

**Table 4:** Medical and surgical treatments in bilateral facial palsy

Treatment	N° pts (%)	Specific treatment	N°of patients
Medical	125 (74%)	Steroid	73
		Antibiotics	51
		Antiviral	20
		Other	27
		Endovenous Ig	23
Surgical	6 (3.5%)	Monolateral facial nerve decompression	3
		Bilateral facial nerve decompression	3
		Mastoidectomy with VT	2
		Mastoidectomy without VT	0
Medical + Surgical	9 (5%)	Bilateral ventilation tube	3
		Bilateral mastoidectomy	2
		Bilateral decompression transmastoid	1
		Monolateral decompression transmastoid	2
		Myringotomy and mastoidectomy	2
Rehabilitation	13 (7.7%)		13

*Ig* immunoglobulins, *VT* ventilation tube

As regards the outcome of the paralysis at last follow-up, in most cases complete bilateral recovery was observed (102/169, 60.4%), with most patients improving their condition within 3 months from its onset. Table 5 reports patterns and timing of recovery from BFP.

**Table 5:** Patterns and timing of recovery from bilateral facial palsy in the 169 patients considered for data analysis

Patterns of recovery	N° pts (%)
Bilateral complete recovery	102 (60.4%)
Bilateral partial recovery	56 (33%)
Complete recovery on one side, partial recovery on opposite side	12 (7%)
Partial recovery on one side, no response on opposite side	2 (1.2%)
No recovery	4 (2.4%)
Death	3 (1.8%)

Timing of recovery	N° pts (%)
Within 30 days	50 (29.6%)
Between 1 and 3 months	45 (26.6%)
Between 3 and 6 months	22 (13%)
Between 6 months and 1 year	15 (9%)
After 1 year	5 (3%)
Not reported	30 (18%)
Death	3 (1.8%)

## Discussion

BFP is an extremely rare clinical finding for a physician. Incidence rate of Bell’s palsy has been estimated around 23–25 per 100.000 persons per year [6, 7] with the rate of simultaneous BFP being about 0.3–2.0% of the total [8]. This condition can often benefit from specific treatment if a proper diagnostic work-up is followed. An aetiological diagnosis was formulated in more than 80% of BFP cases, according to the present review. On the contrary, in unilateral facial palsy (UFP) the vast majority of causes remains unknown, defining this pathology as idiopathic or Bell’s palsy (60–75% or UFP cases [9]). Another difference is the sex prevalence, with males outnumbering females in BFP [10]. However, other characteristics such as age presentation are not different when compared to UFP, which also has a peak incidence between the second and fourth decades [10].

As far as aetiology is concerned, according to the analysed literature, the Authors suggest a modification of the classification by Price T et al. [11], including a total of 9 categories, namely in decreasing order of prevalence: autoimmune, infectious, traumatic, idiopathic, miscellaneous, neoplastic, iatrogenic, metabolic and vascular.

Autoimmune BFP constitutes the most frequent aetiology. This category includes different entities, such as vasculitis (i.e. Wegener granulomatosis), Sjogren’s syndrome, Guillain–Barré syndrome (GBS), Melkersson–Rosenthal syndrome, etc.…Other autoimmune pathologies have a less evident clinical presentation, and the diagnosis may require time, in these cases help comes from autoimmune laboratory tests. For example, particularly difficult is the diagnosis of Melkersson–Rosenthal syndrome, which requires a labial or cheek biopsy during granulomatous phase. Sarcoidosis is an immune-mediated pathology with formation of non-necrotizing granulomas that can affect every organ or tissue. Diagnosis is generally difficult given the variability of clinical presentations and symptoms [12].

Infectious BFP represent the second most frequent etiological category, with the commonest infectious agent being *Borrelia burgdorferi* which causes Lyme’s disease, followed by *HIV* and *HEV*. Less common agents are viruses as *HSV*, *JEV* and *enterovirus*, bacteria as *Clostridium botulinum, Ehrlichia chaffeensis, Leptospira interrogans, Micobatterium Tubercolosis* or fungi as *Cryptococcus* and *Toxoplasma genus*.

Recently, few case reports described a BFP onset after COVID-19 infection or vaccination. Andreozzi et al. [13] described two cases of BFP with paresthesias, both occurring after the first dose of COVID-19 vaccine Vaxzevria^™^, with favorable outcome. The Authors considered the bilateral palsy as a consequence of a GBS. Moreover, five case reports on BFP during COVID-19 infection, were described: two were considered as bilateral Bell’s palsy after COVID-19 [14, 15] in one a mixed infection with COVID-19 and EBV was detected [16] the last two were thought to be variants of GBS [17, 18].

Traumatic BFP is the easiest to evaluate, being the history of head trauma the main element that leads to diagnosis together with other cranial nerves palsy and neurological damages.

Neoplastic pathologies represent only 5% of all cases included by present review, the most common causing pathologies being leukaemia and lymphoma (65% of all neoplastic causes). Therefore, brain MRI for detection of solid neoplasm or meningeal involvement, a complete blood count and blood smear, and sometimes a CSF analysis, must be performed to rule out neoplastic aetiology.

Idiopathic aetiology represents only 16% of the analysed cases, even if the frequency of idiopathic BFP may be even lower.

The flow-chart (Fig. 2) on the work-up of BFP conveys the findings from this literature review, in an attempt to guide physician from any medical field to correctly frame the patient presenting with BFP and possibly increase the chance of recovery through the prompt delivery of the appropriate treatment. Several possibilities regarding the diagnosis can be speculated by careful collection of the medical history, particularly regarding other symptoms onset, which are frequent in BFP. First, the diagnosis of iatrogenic BFP is straightforward and an accurate patient history with previous medical data review is sufficient to lead to diagnosis. Second, the patient should be asked regarding recent head trauma. In this case, after head CT including temporal bone and temporo-mandibular joints, both ENT and neurologic evaluations must be performed. In doubtful cases or in case of additional neurological symptoms, attention should be paid to the intracranial compartment, by performing a brain MRI.

Patients without a history of head trauma or iatrogenic BFP, must be interviewed about recent travels, risky behaviours, tick bite, autoimmune, previous known autoimmune, metabolic and oncologic disorders. Afterwards, a complete physical exam, blood tests and autoimmune tests should be performed in all cases. If neurological signs and symptoms are present, neurology consultation is needed and CSF analysis required according to the specialist consultation. Serology for common infectious diseases that can cause BFP (see Table 1) should be ruled out in all patients and if infectious screening is positive, proper medical treatment must be administered. ENT consultation is recommended in all cases especially to investigate vestibulo-cochlear function.

Since some pathologies (such as neoplasm or temporal bone infections) may present with rapidly progressive symptoms and lead in short time to a poor prognosis, in our opinion it is necessary to perform all cited exams in all patients with no associated or nonspecific symptoms. When all the above-mentioned work up is inconclusive, before diagnosing a BFP as idiopathic, we suggest performing at least a contrast-enhanced brain MRI, which could exclude neoplastic or vascular disorders. A better understanding of the causes leads to a high rate of recovery from BFP (complete bilateral recovery in more than 60% of cases, with most patients improving within 3 months from the palsy onset).

BFP treatment strictly depends on correct diagnosis. Infectious causes may benefit from antibiotics or antivirals, Bell palsy or autoimmune causes may need steroids or Immunoglobulins. Additionally, regardless of specific aetiology, physical therapy by a speech therapist or a physiotherapist can be effective in helping patients to recover strength and relearn specific movements such as eye closure and smile, limiting long term impairment such as the development of synkinesis. When paralysis is congenital or results from physical disruption (i.e., traumatic or iatrogenic) of the facial nerve, or when conservative treatment fails, surgical intervention may be warranted. The dominant concern in upper facial paralysis is impaired eye closure, which can predispose the eye to corneal exposure and threaten vision. In the lower face, loss of oral competence and the ability to smile are primary concerns. Notably, the role of facial nerve decompression in acute stages for patients with complete idiopathic or posttraumatic paralysis (e.g., in Bell palsy and Melkersson–Rosenthal syndrome) is gaining increasing importance [19].

This review is not without limitations. First, literature is scarce of large cohorts of BFP patients, most published cases are case reports, with few retrospective case series usually limited to one specific aetiology. Moreover, studies frequently do not report the length of follow-up and the time between onset and best recovery grade of facial function. Also, there is a lack of data regarding the grading of BFP, both at the onset and at follow-up. Parallel to this, even when reported, the scoring systems used were not uniform. Considering the need for a common language between authors, to compare different studies and patients, the Authors suggest that facial function should always be graded through one, or better two, international grading systems, such as the House–Brackmann grading system and the Sunnybrook grading system [20].

## Conclusion

BFP is a disturbing condition for the affected patient and contrary to UFP, it is generally attributable to a specific cause. Associated aetiologies are various and differential diagnosis is challenging. If a correct diagnosis is formulated, possibilities to recover are elevated and directly correlated to the initiation of appropriate treatment. Physicians should rule out all possible causes before claiming a BFP as idiopathic.
